# Exploring the Patients' Perspective on Digital Tools for Psychosocial Assessment in Dentistry

**DOI:** 10.1111/joor.13909

**Published:** 2025-01-27

**Authors:** Axel Kutschke, Bellarita Bechmann, Birgitta Häggman‐Henrikson, Anders Wänman, Justin Durham, Anna Lövgren

**Affiliations:** ^1^ Department of Orofacial Pain and Jaw Function, Faculty of Odontology Malmö University Malmö Sweden; ^2^ Department of Orofacial Pain and Jaw Function Gävle County Hospital, Public Dental Health County Council of Gävleborg Gävle Sweden; ^3^ Centre for Research and Development Uppsala University/Region Gävleborg Gävle Sweden; ^4^ MedAi.ai GmbH Frankfurt/Main Germany; ^5^ Department of Odontology, Faculty of Medicine Umeå University Umeå Sweden; ^6^ School of Dental Sciences Newcastle University Newcastle UK; ^7^ Newcastle Hospitals NHS Foundation Trust Newcastle UK

**Keywords:** dental care, facial pain, qualitative research, questionnaires, telemedicine, temporomandibular joint disorders

## Abstract

**Background:**

Psychosocial screening is a valuable part of the assessment of patients with orofacial pain, as psychosocial factors will affect prognosis and treatment outcomes. Paper‐based questionnaires are predominately used to assess the degree of psychosocial comorbidity; however, digital alternatives for screening questionnaires may be more cost‐effective and resource‐saving if patients are receptive to using them.

**Objective:**

To evaluate how patients perceive digital psychosocial screening in dentistry.

**Method:**

Using a qualitative approach, individual semi‐structured interviews were conducted with a purposive sample of adult patients with orofacial pain (*n* = 16) recruited from specialist dental clinics in Umeå and Gävle, Sweden. The interviews were transcribed verbatim and then analysed using Qualitative Content Analysis. Before the interviews, patients first completed the paper‐based questionnaires and then the digital version.

**Results:**

The analysis of patients' experiences resulted in an overarching theme: Patients appreciate a holistic approach, thus valuing psychosocial screening, and they particularly favour screening in a digital format. From this theme, two categories emerged:

1. Perceptions about health shape patients' expectations of dental care, and with deeper understanding of the value of psychosocial assessment, patients appreciate a holistic approach that includes psychosocial factors.
2. Digital screening is perceived by patients as a reliable, meaningful and environmentally sustainable method.

**Conclusions:**

In general, the patients appreciated a holistic approach in dentistry and understood the value of psychosocial screening as part of this. From the patients' perspective, digital psychosocial screening was both acceptable and beneficial. The findings support the introduction of digital psychosocial screening into daily dental practice.

## Graphical abstract

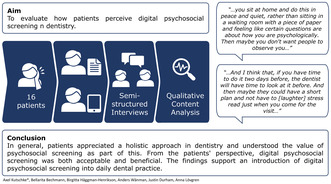

## 1 Introduction

eHealth is defined as ‘the cost‐effective and secure use of information and communications technology in support of health and health‐related fields’; however, it is still driven by assumptions from care providers about its benefits rather than evidence of its applicability [1]. The adoption of eHealth services is influenced by various factors, including cost, patient value, privacy and security concerns. Previous analyses conducted in the context of eHealth research have not focused on these factors, which leads to questions about the credibility and accuracy of eHealth due to a lack of evidence [2]. This can result in the reluctance of policymakers to invest in and develop policies related to the implementation of eHealth [3].

Although eHealth is becoming an increasingly relevant part of healthcare and is now available in a wide range of healthcare services, patient questionnaires in dentistry today are still mainly paper‐based despite their disadvantages and limitations [4, 5]. Comparisons between digital and paper‐based questionnaires show that, while the data quality and reliability of both methods are comparable, health professionals nevertheless perceived digital systems as less suitable for certain patient groups and more complicated to implement [6, 7, 8].

Effective management of chronic diseases in healthcare requires a nuanced understanding of the factors that influence prognosis, treatment adherence and treatment outcomes [9]. A standardised psychosocial assessment has therefore been recommended for new adult patients in general dentistry and has been shown to prevent unstructured and biased clinician‐specific assessments [10, 11]. For orofacial pain patients, screening for psychological comorbidity is important to help tailor treatment to their psychosocial profile [12]. The most common type of chronic pain in the orofacial region is temporomandibular disorders (TMD), a group of musculoskeletal disorders that cause pain or functional limitation or both in the jaw muscles and the temporomandibular joint (TMJ) and its associated structures [13]. The prevalence of TMD is approximately 10% of the population, with twice as many women affected as men, and is associated with a profound impact on daily life [14, 15, 16]. TMD is associated with several psychosocial comorbidities such as stress, depression, anxiety, physical symptoms and catastrophising. Due to these psychosocial comorbidities, TMD should be evaluated from a biopsychosocial perspective [17].

This multidimensional and biopsychosocial perspective is reflected in the current diagnostic system for TMD, the Diagnostic Criteria for TMD (DC/TMD) [13]. The DC/TMD provides both a standardised and valid physical diagnosis (Axis I) and evaluates psychosocial factors that might influence treatment and prognosis (Axis II). Axis I is based on physical and biological aspects of the clinical characteristics, whereas Axis II evaluates the psychosocial aspects by assessment of psychosocial and behavioural factors with reliable and valid questionnaires [13]. The digitaliation of the comprehensive Axis II is therefore the next logical step in the implementation of eHealth in dentistry. However, implementation requires, among other factors, acceptance by both care providers and patients [18]. In this context, the patients' experiences of using digital questionnaires in general, and specifically for psychosocial screening in dentistry, remain unexplored.

The aim of this study was, therefore, to evaluate patients' perspectives of digital psychosocial screening in the context of dentistry.

## 2 Methods

### 2.1 Study Design

This study was conducted in 2022–2023 in the regions of Västerbotten and Gävleborg in Northern Sweden. Both regions have approximately 280 000 inhabitants each, 60% of whom (aged 23 years and older) reported to have visited their dentist at least once during 2019–2021, with the majority attending the Public Dental Healthcare Services clinics [19].

We used the experimental web‐based software, DIGITAL DC‐TMD (MedAi.ai GmbH), which included the comprehensive Axis II in the DC/TMD, complemented by the Perceived Stress Scale (PSS‐10) and the Pain Catastrophising Scale (PCS).

### 2.2 Questionnaires

The Graded Chronic Pain Scale v2 (GCPS) is a short (8‐item), reliable and valid instrument that assesses pain intensity and pain‐related disability [20]. The Jaw Function Limitation Scale (JFLS‐20) consists of 20 items and assesses global limitations across mastication, jaw mobility and emotional and verbal expression [21]. The Oral Behaviours Checklist (OBC) is a 21‐item‐based instrument used to identify and quantify the frequency of oral parafunctional behaviours [22]. The Patient Health Questionnaire (PHQ‐9) is used for assessing the severity of depression based on the Diagnostic Criteria for Major Depressive Disorder [23]. The Generalised Anxiety Disorder Screener (GAD‐7) assesses generalised anxiety disorder, validated in a general population [24]. The Patient Health Questionnaire (PHQ‐15) consists of 15 items assessing somatic symptom severity [25]. PSS‐10 consists of 10 items that evaluates feelings and thoughts during the past month to assess the degree to which situations in one's life are appraised as stressful [26]. PCS assesses, with 13 items in three subscales (rumination, magnification and helplessness), catastrophising thoughts and the related behaviour [27].

All participants were patients referred to specialist dental clinics in Umeå and Gävle. An analog version of the complete set of instruments was mailed to the patients' home addresses, prior to the examination appointment at the dental clinic. After the examination, patients who provided consent to participate in the study were emailed a link to the digital version of the questionnaires. Upon completion of the digital questionnaires, the participants were contacted by telephone to schedule an interview. The interviews were conducted in two different settings, live in a meeting room at the clinic or online via Zoom, at a time preferred by the participants. The individuals conducting the interviews were not involved in the care of any of the participants and had no prior interaction with them. The study was approved by the Regional Ethical Review Board of Umeå University (2022–00110‐01) and conducted in accordance with the Ethical Principles for Medical Research Involving Human Participants (Declaration of Helsinki). All participants participated on a voluntarily basis without any remuneration and were guaranteed confidentiality, meaning they would not be identifiable in publications. They were informed about their right to withdraw their participation without providing a reason until the time of publication of the study. Written informed consent was obtained from all participants.

### 2.3 Data Collection

**FIGURE 1:**
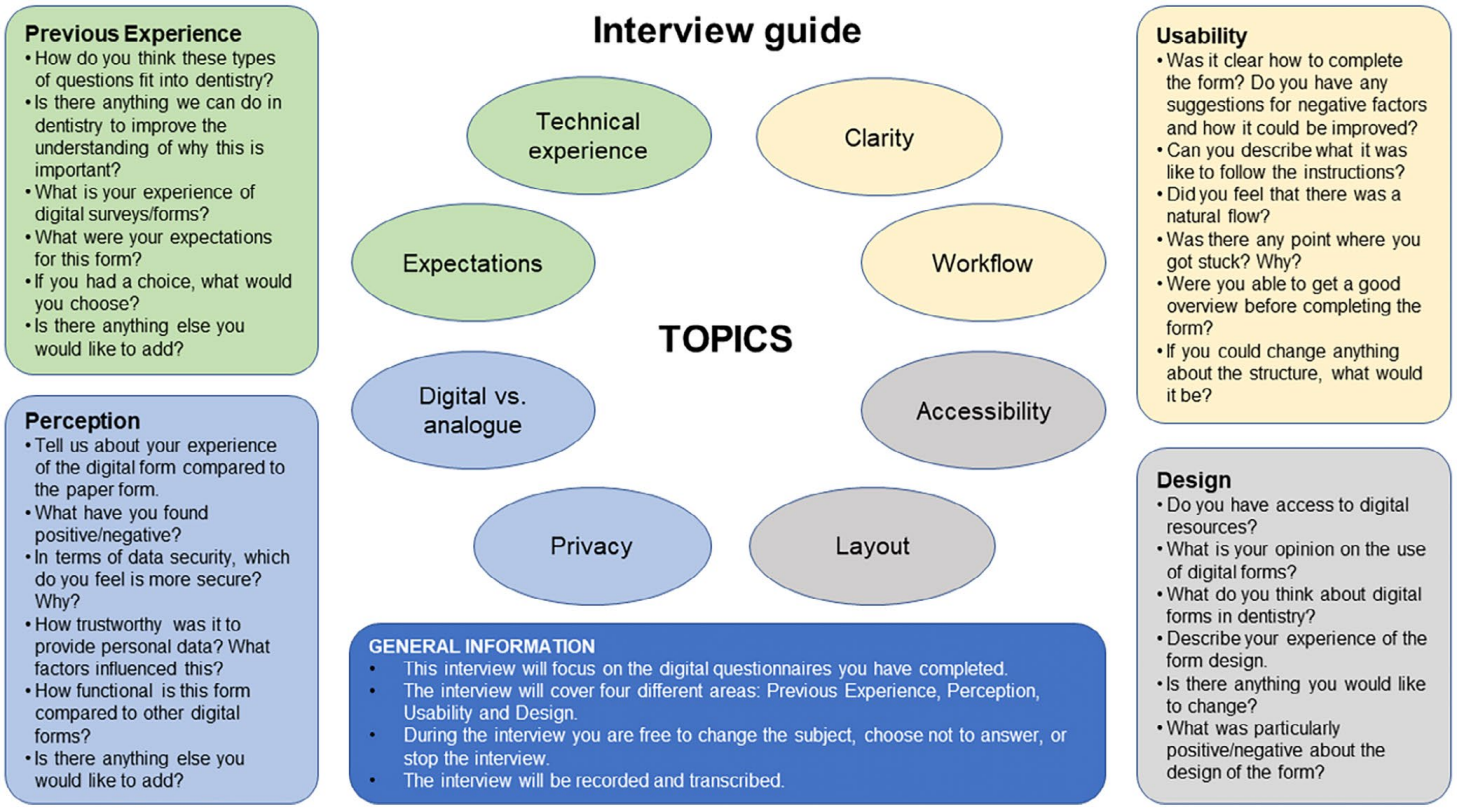
Graphical interview guide illustrating the targeted topics during the interviews.

Individual semi‐structured interviews were conducted by three undergraduate dental students and two orofacial pain/TMD specialists (A.L., A.K.), all of whom were trained in interview techniques and involved in developing the interview guide. The interviews were designed so that the respondents could answer without restrictions, allowing additional aspects to be introduced by the respondents and the interviewer. A pre‐tested, semi‐structured graphical interview guide (Figure 1) was developed to allow structured and open‐ended questions. The participants were asked about psychosocial screening in dentistry covering four major categories: Previous experience, Perception, Usability and Design. The graphical interview guide covered these categories together with specific questions and was used as a support tool for the interviewer in adding probing and follow‐up questions during the interviews in adding probing and follow‐up questions that require more specific answers, for example, ‘What makes you think that?’ and ‘What factors have affected your choice?’ We conducted a pilot study with four interviewees to explore the functionality of the digital questionnaire's platform and the interview guide. No issues were identified; therefore, no changes were required to the interview guide. Each interview lasted approximately 30 min; all interviews were audio‐recorded and transcribed verbatim. Transcripts were checked by one of the authors (A.K.) against the recordings and adjusted as needed.

### 2.4 Data Analysis

The interviews were analysed using Qualitative Content Analysis with an inductive approach [28]. The transcribed data were then coded independently by two co‐authors (A.L., A.K.). Each transcript was read repeatedly as a whole and in parts during data analysis. Thereafter, the text was divided into meaning units and phrases, and phrases relevant to the research question were identified and sorted into condensed meaning units and assigned codes. The codes were repeatedly compared and interpreted to find similarities and differences. In addition, the codes, subcategories and categories were analysed at a manifest (close to text) level, whereas the overarching theme included interpretations that corresponded to the latent meaning of the material. Two co‐authors (A.L. and A.K.) conducted the analysis in parallel, and the findings and interpretations were compared and discussed until agreement was reached. To increase trustworthiness and credibility, the results were discussed in the research group (A.K., A.L., B.H.‐H., A.W., J.D. and B.B.) to minimise subjective interpretation [29]. The SRQR guidelines were followed (Appendix 1, [30]).

The results are presented with examples of representative quotes from the interviews, subcategories, categories and an overall theme.

## 3 Results

In total, 16 adults (8 women and 8 men) aged 25–66 years were recruited among patients referred for assessment to the Department of Clinical Oral Physiology, Umeå and the Department of Orofacial Pain and Jaw Function, Gävle (Table 1).

**TABLE 1:** Characteristics of the study population (*n* = 16).

	Gender (Women/Men)	Median age (range years)	Geographical area (Gävle/Umeå)
Patients	(8/8)	46 (25–66)	(5/11)

The analysis resulted in one main theme, two categories and seven subcategories (Table 2). The theme—Patients appreciate a holistic approach, thus valuing psychosocial screening, and they particularly favour screening in a digital format—highlights the patient's appreciation and positive experiences from using digital questionnaires for psychosocial screening.

**TABLE 2:** Main theme, two categories and seven sub‐categories summarising the study findings.

Patients appreciate a holistic approach, thus valuing psychosocial screening, and they particularly favour screening in a digital format	
Perceptions about health shape patients' expectations of dental care, and with deeper understanding of the value of psychosocial assessment, patients appreciate a holistic approach that includes psychosocial factors	*It is difficult to quantify problems*, *but clear questions and the use of scales make it easier*
	*Dental patients' uncertainties about knowledge of mental health illnesses mean that*, *for the patient to appreciate them as relevant*, *the questions must be contextualised*
Digital screening is a trustworthy and meaningful measure for the patient	*Effective and expedient flows are perceived positively but also create expectations*
	*Questionnaires need to be clear*, *but flexibility is appreciated*
	*Individual abilities are identified as potential obstacles*
	*Patients perceive digital questionnaires as sufficiently secure*
	*Design is an important element for establishing credibility*

In the following, the two categories are presented in bold, and their corresponding subcategories are in italics.

**Perceptions about health shape patients' expectations of dental care, and with deeper understanding of the value of psychosocial assessment, patients appreciate a holistic approach that includes psychosocial factors**.

This category consists of two subcategories:

1. *It is difficult to quantify problems*, *but clear questions and the use of scales make it easier*.
2. *Dental patients' uncertainties about knowledge of mental health illnesses mean that*, *for the patient to appreciate them as relevant*, *the questions must be contextualised*.

In general, the questionnaires received positive feedback from the patients, who appreciated its comprehensiveness, ease of use and clear questions rather than free text responses.

It was quite extensive. It was much more (extensive) than I had thought it would be. But it was rather straightforward anyway. (Male, 42 years old, P11)

However, it proved difficult to accurately assess subjective experiences and to recall symptoms retrospectively.

I have filled in forms before where you had to write a lot. I think it's good that you only need to click on a scale. In other words, there is not so much free text. (Female, 37 years old, P6)

‘How many days in a six‐month period have you had pain?’, for example. Eh. It's quite difficult to answer. I think it would be easier to answer (the question) ‘How many days in a week (approximately) have you had pain?’ or something like that. But still, even that would be quite difficult (to answer). (Female, 37 years old, P6)

Patients are unsure about how knowledgeable dental practitioners are about mental health illnesses. Therefore, the questions needed to be put into context for the patient to view them as relevant to dental care. However, in general, the questions were perceived as relevant to dental care.

I think it actually fits (pause) quite well. They were especially good questions, sure. Sure, I had some thoughts about the questions and the connection. They were also about stress and mental health, and things like that. But you also know that these affect all areas. I thought it was very interesting that it was rather broad. (Female, 42 years old, P4)

Additionally, many patients expressed a desire for an explanation regarding the connection between mental health illness and dental treatment.

So, the expectation was that it would be a little … less cumbersome. Now, it wasn't, and that's where I think it will connect to the fact that if you can describe why I should fill in these questions and what the connection is to my mental health in the form of stress or irritation or something like that, it won't feel as cumbersome because then you understand the purpose behind it. (Male, 28 years old, P16)

Lastly, it is worth noting that the patients express that there is still a sense of shame surrounding psychological issues in Sweden.

I think that you'd feel safer if, like me, you sit at home and do this in peace and quiet rather than sitting in a waiting room with a piece of paper and feeling like certain questions are about how you are psychologically. Then maybe you don't want people to observe you, and such. We still are somewhat shameful today about certain things in Sweden. (Female, 42 years old, P4)

**Digital screening is a trustworthy and meaningful measure for the patient**.

This category covers aspects of trustworthiness in the digital version of the questionnaires and the importance of efficient and effective flows through the forms and views on how secure users feel about their data when completing the form.

The category comprises of five subcategories:

*Effective and expedient flows are perceived positively but also create expectations*.

The patients expressed that completing the questionnaires at home was convenient and saved travel time, benefiting both themselves and the environment.

And like I said, for those who live far away, ‘This is great. It is superb!’ Yes, it saves a lot of money. (Male, 45 years old, P9)

So, I think it's good. For one thing, we are saving forests, and such. I think we should think about the future. (Female, 54 years old, P3)

Additionally, patients described that the risk of paperwork being misplaced or lost in the mail was eliminated.

It has become a bit of a hassle with mail now too; (for example,) when you have to find a mailbox and mail things back, (but) you can forget them in the car, and stuff like that. (Female, 42 years old, P4)

The patients expressed that the digital format of the questionnaires also allowed for perceived streamlining of the data‐collection process.

If you say that it (the results of the questionaries) goes into my journal, that means that it is there; in other words, it goes all the way (i.e., is thorough). (Female, 42 years old, P4)

Patients shared their optimism about shorter durations of patient management when the digital forms were used, contributing to their high expectations of efficiency.

There are many advantages. The processing could hopefully be shorter. (Female, 65 years old, P15)

Moreover, some patients thought that the availability of their information from the questionnaires before the dental visit enabled the dentist to be better prepared.

And I think that, if you have time to do it two days before, the dentist will have time to look at it before. And then maybe they could have a short plan and not have to (laughter) stress‐read just when you come for the visit. (Female, 37 years old, P6)

*Questionnaires need to be clear*, *but flexibility is appreciated*.

The patients found it easier to change their answers when they answered the questions digitally.

… it is easier to erase than to write with a pen. So, it's easier. (Female, 54 years old, P3)

In addition, the digital format made it less likely to skip or overlook questions compared to paper forms.

In a paper form, it is easy to skip a step. Or to forget the back side of the paper. Here it goes step by step. There will be better flow in a digital form. (Female, 47 years old, P12)

Patients also reported feeling stressed when filling out the paper forms in the waiting room.

And it's not quite as much pressure as when you're sitting in the waiting room. (Female, 37 years old, P6)

And it's just that I can do it on the bus on the way home. Or if I'm sitting at home in peace and quiet, I can do it then. Not, as they say, in the waiting room right before you go in so that when you're done, there are lots of people. When you feel that you can now take the time (pause), you are calm and harmonious, so to speak. (Female, 42 years old, P4)

Overall, patients expressed a desire for an overview and summary of their answers after completing different parts and before submitting.

I wonder if you could go back and see what you had answered for certain questions. I don't know if that is possible. Or that you get some kind of compilation. Because when you had answered a partial form, for example, then it disappeared. (Female, 47 years old, P12)

Some patients mentioned that paper forms provided a better overview, whereas others emphasised the importance of a clear overview and instructions, as well as the ability to navigate back and forth, which was perceived as facilitated in the digital format.

Paper forms are more manageable. Because then you see everything. And it is much faster too. (Female, 50 years old, P8)

It should be easy to understand. Yes, type one, click here, start, etc. And then it becomes a continuous flow instead of having to choose parts to respond to. (Female, 47 years old, P12)

*Individual abilities are identified as potential obstacles*.

Patients expressed that an individual's thoughts about their own abilities and the abilities of others, as well as prejudices related to age and interest in technology, can also influence their willingness to answer the digital version of the questionnaire.

It's very subjective evidence in the form of people close to me, well, my grandmother and grandfather, who were born in the forties, and they've digitized anyway, so it wouldn't be a problem for them. But what if you take one? Let's say they have a friend who hasn't digitized, well then, it's a problem because they might not have access to an email address or a computer in the same way or a smartphone. But I still think most of the older people you see around you when you walk around town, they have access to a smartphone. (Male, 28 years old, P16)

*Patients perceive digital questionnaires as sufficiently secure*.

Patients conveyed that they felt confident to complete the digital version of the questionnaire and to submit it on the website. According to our participants, the healthcare system in Sweden is in general regarded a trustworthy system that provides accurate and secure management of user data.

But I trust that what they (Swedish healthcare system) get from you is properly taken care of. So, I personally have no problem with that. (Male, 25 years old, P5)

Yeah, I don't worry about that then. I think it's GDPR everywhere. (Female, 37 years old, P6)

Now I don't think that it is very sensitive information, so to speak; so, I have no problem with it somehow leaking out. It's not something I'm worried about like my bank accounts. (Male 62 years old, P10)

Oh, I expect it to be as reliable as any computer system. I don't see any difference there, in the healthcare sector as a whole. (Male, 55 years old, P1)

*Design is an important element for establishing credibility*.

Regarding the digital version of the questionnaires, patients indicated that the form, content and structure were found to be comparable to questionnaires used by other well‐known and reputable websites or research institutions.

Yes, they are quite similar I think (compared with 1177.se is the Swedish healthcare system’s dedicated website. It is also a telephone helpline for patients and the tax authority). There was no difference. Simple, straightforward, nothing complicated. Just the question and then you have to tick the answers. They are quite similar in that way. (Female, 50 years old, P14)

## 4 Discussion

To our knowledge, this study is the first to investigate how patients experience digital psychosocial screening in dentistry. Our findings show that, in general, the patients' experiences of digital psychosocial screening were positive and emphasise the importance of a holistic approach in dentistry. The patients' initial expectations were influenced by their general health perceptions, which were based on their understanding of health, which includes physical health, mental health and social well‐being. However, as the patients gained a deeper understanding of the value of psychosocial assessment, they appreciated a holistic approach that takes psychosocial factors into account in the clinical decision‐making process.

The patients recognised their individual abilities, age‐related differences and varying level of interest in technology as potential obstacles to completing the digitalization gap between older and younger generations. Moreover, concerns about who may lack access to the relevant technology and how the perception of individual abilities and prejudices may influence patients' willingness to engage with the digital version outweighed factors such as gender, household type and place of residence [31, 32].

Patients appreciated the convenience of completing questionnaires at home, emphasising the benefits of time saving, particularly for those living far away [33]. The elimination of risks associated with traditional paper questionnaires, such as misplacement or loss in the mail, emerged as a notable advantage of the digital format. Patients expressed relief from the hassle of dealing with physical forms, aligning with a broader trend toward reducing the reliance on paper‐based processes. As mentioned, this could be one of the main factors for higher response rates in web‐based questionnaires [34]. Patients even expressed an expectation that the use of digital questionnaires could lead to the dentist being better prepared before each visit since the information would be available already before the intake examination. Such expectations from the patients would need to be carefully managed by the dental practitioners. This would in turn require time for preparation ahead of each visit which may be challenging in general dental practice but probably more feasible in specialised clinic settings. Moreover, the patients showed an environmental consciousness, which calls for a broader societal perspective on sustainable practices in health care.

The long‐standing stigma surrounding psychological issues was acknowledged by patients, and this influenced their preferences for digital screening to be conducted in private. Other studies have shown that digital questionnaires captured more sensitive behaviours and showed fewer missed questions than paper‐based questionnaires [35].

The patients were confident that completing the questionnaires digitally was safe. They were also positively inclined to share health information in general, particularly information with psychosocial aspects. They cited trust in the Swedish healthcare system, which is an important factor for patients' adherence to eHealth services and surprisingly did not consider the information to be particularly sensitive [36]. They expressed familiarity with General Data Protection Regulations (GDPR) which contributed to the sense that their data were secure.

The similarity of the digital version of the questionnaire with other well‐known healthcare and governmental websites, in terms of both the design and the structure, contributed to the patients perceiving the questionnaire as trustworthy and valid. The presentation and format of the questions were, as noted in a study on completing Digital Quality of Life Questionnaires, a common theme discussed by the patients and cited as a reason for the delay in completion time [37]. Furthermore, poor design or structure of eHealth applications has been identified as a significant usability barrier [38]. In our study, the patients expressed the desire for comprehensive, easy‐to‐use questionnaires over free‐text responses and emphasised the importance of a user‐friendly interface in enhancing the overall experience. However, difficulties arose in accurately rating subjective experiences, quantifying problems and recalling past information.

Whereas existing literature acknowledges the importance of clear and comprehensible questions in digital health tools, this study adds a more nuanced perspective [2]. Patients express a desire for context and explanation, especially regarding the relationship between psychosocial factors and dental treatment. This suggests a need for explanatory components in psychosocial screening questionnaires, whether paper‐based or digital. Providing additional context in the questionnaire to improve patients' understanding of the importance of the questions could lead to more truthful answers on the questions, increasing the validity of the outcomes. On the other hand, such additional information may contribute to potential bias in the outcomes, and additional research would be needed to further investigate this. Nevertheless, these findings indicate the importance of including information about the importance of psychosocial screening in dentistry at least at the intake examination.

Implementing digital psychosocial screening into routine dental care depends not only on patient acceptance but also on challenges related to technological infrastructure, including limited access and digital literacy among both providers and patients [39]. In this regard, the use of digital solutions should incorporate sufficient security to protect sensitive information from outside attack and technical malfunctions. There may also be compatibility issues when integrating digital methods in already existing electronic health record systems. From a practitioner's perspective, resistance to change may pose a barrier, as dentists may be hesitant to incorporate digital psychosocial screening into their established workflows [40]. Adequate training becomes imperative to familiarise practitioners with the use and interpretation of digital psychosocial screening tools.

### 4.1 Strengths and Limitations

To allow for both the depth and breadth of information available for analysis, participants of different ages and genders were included. The individual interviews were analysed with Qualitative Content Analysis (QCA). QCA has shown to be a systematic method of analysing written or verbal communication and is often used in analyses of people's experiences and reflections. QCA focuses on the differences between and similarities within codes and categories. The method allows for both manifest and latent interpretations of the content; however, the interpretations may vary in depth and level of abstraction [28].

Trustworthiness was achieved by fulfilling the aspects of credibility, confirmability, dependability and transferability [29]. To ensure credibility, triangulation—the process of using multiple perspectives to strengthen the credibility of the findings—was used between the researchers, and several interpretations were discussed and negotiated before agreeing on the findings [29]. The different backgrounds (i.e., general dental practice, specialist dental practice, clinical and healthcare management and dental education in different European contexts) and levels of expertise (i.e., TMD, orofacial pain, epidemiology and qualitative methods) of the authors influenced the data analysis and enriched the triangulation. Quotations and descriptions of the study context were used to enhance the confirmability of the findings. Data were collected and analysed consistently to create dependability. Our results should be considered in other contexts to ensure transferability, but we consider our findings representative of patients visiting special dental care clinics in Sweden.

The dental background of the interviewers was identified as a possible risk, as it could make participants feel uncomfortable about sharing their opinions. However, strategies were taken to counteract such an effect. One strategy was to conduct the interviews in a non‐clinical setting and another was to not involve the interviewers in the assessment or treatment of the study participants [41].

Furthermore, the study population had a relatively high level of experience with the use of digital questionnaires, which may have had a positive impact on the results. In addition, an even wider age distribution could have provided a more applicable result.

## 5 Conclusions

Our study suggests that digital psychosocial screening is a meaningful tool in the context of dental care, particularly in the assessment and the management of patients with chronic orofacial pain and jaw dysfunction. Considering both the advantages and the disadvantages, we believe that incorporating digital questionnaires into contemporary dentistry could be of great benefit. Future research in this area is necessary to develop and evaluate digital solutions that can meet the expectations of both users and practitioners. The patients' reported experiences expressed a positive attitude towards psychosocial screening in dentistry in general and to digital screening in particular, which should pave the way for the implementation of digital psychosocial screening in everyday care.

## Author Contributions

A.K., A.L., B.B. and B.H.‐H. designed the study. A.K. and A.L. collected the data. A.K. and A.L. analysed the data. B.H.‐H., A.W. and J.D. reviewed and discussed the findings. A.K. drafted the manuscript. A.K., A.L., B.H.‐H., A.W., B.B. and J.D. critically reviewed and approved the final manuscript.

## Ethics Statement

The study was approved by the Regional Ethical Review Board at Umeå University (2022–00110‐01) and was carried out in accordance with the Helsinki Declaration. Informed consent was obtained from the participants.

## Consent

The authors have nothing to report.

## Conflicts of Interest

The authors declare no conflicts of interest.

### Peer Review

The peer review history for this article is available at https://www.webofscience.com/api/gateway/wos/peer‐review/10.1111/joor.13909.

## Data Availability

The data analysed in the current study are available from the corresponding author on reasonable request.

## Appendix 1

**Table:**

	**Standards for Reporting Qualitative Research (SRQR)***	
	http://www.equator‐network.org/reporting‐guidelines/srqr/	
		**Page/Line no(s)**
**Title and abstract**		
	**Title:** Concise description of the nature and topic of the study Identifying the study as qualitative or indicating the approach (e.g., ethnography, grounded theory) or data collection methods (e.g., interview, focus group) is recommended	1/1–2
	**Abstract:** Summary of key elements of the study using the abstract format of the intended publication; typically includes background, purpose, methods, results and conclusions	2/6–3/12
**Introduction**		
	**Problem formulation**—Description and significance of the problem/phenomenon studied; review of relevant theory and empirical work; problem statement	6/4–7
	**Purpose or research questio**n—Purpose of the study and specific objectives or questions	6/8–9
**Methods**		
	**Qualitative approach and research paradigm**—Qualitative approach (e.g., ethnography, grounded theory, case study, phenomenology and narrative research) and guiding theory if appropriate; identifying the research paradigm (e.g., postpositivist, constructivist/interpretivist) is also recommended; rationale**	9/1–2
	**Researcher characteristics and reflexivity**—Researchers' characteristics that may influence the research, including personal attributes, qualifications/experience, relationship with participants, assumptions, and/or presuppositions; potential or actual interaction between researchers' characteristics and the research questions, approach, methods, results and/or transferability	19/20–24
	**Context**—Setting/site and salient contextual factors; rationale**	6/13–14
	**Sampling strategy**—How and why research participants, documents, or events were selected; criteria for deciding when no further sampling was necessary (e.g., sampling saturation); rationale**	7/15–19
	**Ethical issues pertaining to human subjects**—Documentation of approval by an appropriate ethics review board and participant consent, or explanation for lack thereof; other confidentiality and data security issues	7/24–25
	**Data collection methods**—Types of data collected; details of data collection procedures including (as appropriate) start and stop dates of data collection and analysis, iterative process, triangulation of sources/methods, and modification of procedures in response to evolving study findings; rationale**	8/6–24
	**Data collection instruments and technologies**—Description of instruments (e.g., interview guides, questionnaires) and devices (e.g., audio‐recorders) used for data collection; if/how the instrument(s) changed over the course of the study	8/6–23
	**Units of study**—Number and relevant characteristics of participants, documents, or events included in the study; level of participation (could be reported in results)	9/21–24
	**Data processing**—Methods for processing data prior to and during analysis, including transcription, data entry, data management and security, verification of data integrity, data coding, and anonymization/de‐identification of excerpts	8/22–23
	**Data analysis**—Process by which inferences, themes, etc., were identified and developed, including the researchers involved in data analysis; usually references a specific paradigm or approach; rationale**	9/2–12
	**Techniques to enhance trustworthiness**—Techniques to enhance trustworthiness and credibility of data analysis (e.g., member checking, audit trail, triangulation); rationale**	9/12–14
**Results/Findings**		
	**Synthesis and interpretation**—Main findings (e.g., interpretations, inferences and themes); might include development of a theory or model, or integration with prior research or theory	9/25–10/3
	**Links to empirical data**—Evidence (e.g., quotes, field notes, text excerpts, photographs) to substantiate analytic findings	10–16
**Discussion**		
	**Integration with prior work, implications, transferability and contribution(s) to the field**—Short summary of main findings; explanation of how findings and conclusions connect to, support, elaborate on or challenge conclusions of earlier scholarship; discussion of scope of application/generalizability; identification of unique contribution(s) to scholarship in a discipline or field	16/11–19/4
	**Limitations**—Trustworthiness and limitations of findings	20/4–11
**Other**		
	**Conflicts of interest**—Potential sources of influence or perceived influence on study conduct and conclusions; how these were managed	21/23
	**Funding**—Sources of funding and other support; role of funders in data collection, interpretation, and reporting	21/10–11
	*The authors created the SRQR by searching the literature to identify guidelines, reporting standards, and critical appraisal criteria for qualitative research; reviewing the reference lists of retrieved sources; and contacting experts to gain feedback. The SRQR aims to improve the transparency of all aspects of qualitative research by providing clear standards for reporting qualitative research.	
	**The rationale should briefly discuss the justification for choosing that theory, approach, method, or technique rather than other options available, the assumptions and limitations implicit in those choices, and how those choices influence study conclusions and transferability. As appropriate, the rationale for several items might be discussed together.	
	**Reference**	
	B.C. O'Brien, I.B. Harris, T.J. Beckman, D.A. Reed, and D.A. Cook. “Standards for Reporting Qualitative Research: A Synthesis of Recommendations”. Academic Medicine, 89, no. 9 (2014), https://doi.org/ 10.1097/ACM.0000000000000388	
